# Nutrient intake of young South African adults from the baseline of the African-PREDICT cohort study

**DOI:** 10.1017/S1368980024002076

**Published:** 2024-10-29

**Authors:** Marina Visser, Claudine Jordaan, Edelweiss Wentzel-Viljoen, Aletta Elisabeth Schutte, Tertia Van Zyl

**Affiliations:** 1 Centre of Excellence for Nutrition, North-West University, Potchefstroom 2531, South Africa; 2 MRC Research Unit for Hypertension and Cardiovascular Disease, North-West University, North-West Province, Potchefstroom 2531, South Africa; 3 Hypertension in Africa Research Team (HART), Medical Research Council Unit for Hypertension and Cardiovascular Disease, North-West University, Potchefstroom 2531, South Africa; 4 School of Population Health, University of New South Wales, Sydney, NSW 2052, Australia; 5 The George Institute for Global Health, Sydney, NSW 2042, Australia

**Keywords:** Nutrient intake, Nutrient patterns, Ethnicity, Socio-economic status, Young adults, South Africa

## Abstract

**Objective:**

This study aimed to describe and compare the nutrient intake of young adults in the African Prospective Study on the Early Detection and Identification of Cardiovascular Disease and Hypertension (African-PREDICT) study according to ethnicity and socio-economic status (SES).

**Design:**

Cross-sectional analysis of baseline nutrient intakes in the African-PREDICT study.

**Setting:**

North West Province, South Africa

**Participants:**

Black and white adults (*n* 1153), aged 20–30 years, were classified into three SES groups. Dietary data were collected using three multiple-pass 24-h dietary recalls.

**Results:**

Among all participants, over 70 % failed to meet the estimated energy requirements and the estimated average requirements (EAR) for seventeen of the nineteen reported micronutrients. Across SES groups, more than 50 % of participants consistently did not meet the EAR for Ca, Mg, folate, pantothenic acid and biotin, as well as vitamins A, C, D and E. Participants’ distribution by nutrient pattern tertiles showed high adherence to two patterns: one rich in animal protein and saturated fat, and the other in Mg, potassium, Ca, phosphorus and fibre. This was seen only in white participants and high SES. Black participants and low SES showed higher adherence to a plant protein, B-vitamins, Zn and Fe nutrient pattern.

**Conclusions:**

The dietary intake of young adults in this study was restricted, with none of the groups meeting nutrient requirements for essential nutrients. Further research is needed to establish a direct link between nutrient patterns and the early detection and identification of CVD and hypertension.

CVD, a major subset of non-communicable diseases, are an increasing global threat, with the most significant rise observed in low- and middle-income countries^(1,2)^. According to the WHO, South Africa has one of the highest rates of CVD in the world. In 2017, it was estimated that about 28 % of all deaths in the country were due to CVD, which is significantly higher than the global average of 17 %^(3)^. Diet represents the most important modifiable factor in preventing CVD; specifically plant-based foods showed a protective effect on cardiovascular function and are currently an area of great interest^(4–9)^.

South Africa has experienced rapid urbanisation in recent years^(10)^, leading to its population manifesting various dietary concerns. In the absence of national data on the dietary intake of adults in the country, the evidence for eating habits has depended on localised studies. The latest available dietary study demonstrated that there has been relatively low consumption of fresh nutritious food, as well as a significant increase in the consumption of processed and fast foods, as well as a decrease in the eating of traditional foods^(11,12)^. Consequently, there has been an increase in the prevalence of non-communicable diseases, as well as a rise in the prevalence of micronutrient deficiencies^(13,14)^. In addition, dietary intake studies of ethnic groups have shown that black Africans had monotonous, nutrient-deficient diets^(15–17)^. The Prospective Urban and Rural Epidemiology (PURE-NWP-SA) cohort study (2005, 2010 and 2015) found that micronutrient deficiencies remain widespread among black Africans, although there was some improvement in nutrient intake between 2005 and 2010 according to a longitudinal analysis^(18,19)^.

The South African government has implemented various initiatives to promote healthier dietary intake^(20)^. To address micronutrient deficiencies, the mandatory South African National Food Fortification Programme (NFFP) of staple foods, namely maize meal and bread flour, was established in 2003^(21)^. Additionally, the South African Food Based Dietary Guidelines (SAFBDG) recommend a diverse, nutrient-rich diet to ensure an adequate individual dietary intake, which influences overall nutritional status^(22)^. However, poverty can significantly impact adherence to dietary recommendations and food choices. Socio-economic status (SES) is, therefore, an important determinant of food selection and overall health, including cardiovascular health, and has been recognised as a factor contributing to adverse outcomes^(23–25)^. Individuals with higher economic status tend to consume nutrient-rich foods, whereas lower SES groups may purchase cheaper but nutrient-poor and energy-dense foods^(26,27)^. Moreover, owing to urbanisation and the resulting increase in SES in South Africa, the population’s dietary intake tends to be different across the SES groups^(28,29)^. Current dietary intake data from young adults of different ethnicities in South Africa are limited.

The aim of this study is to report on the baseline nutrient intake and nutrient patterns of the African Prospective Study on the Early Detection and Identification of Cardiovascular Disease and Hypertension (African-PREDICT) study. Dietary intake is an important predictor of developing CVD that is measured in the African-PREDICT study, which makes reporting on the baseline dietary intakes of the participants important.

## Methods

### Study design and participants

The African-PREDICT study is a longitudinal investigation aimed at identifying and understanding the early pathophysiological changes in cardiovascular function, as well as specific predictors which contribute to the development of hypertension in a bi-ethnic sample. The original study African-PREDICT used an ethnicity/age/sex/SES stratified sampling design and includes a total of 1202 participants^(30)^. The participants included are young, apparently healthy volunteers aged 20–30 years, self-reported black and white ethnicity, with a brachial blood pressure of < 140 and 90 mmHg, HIV-uninfected, not pregnant or breast-feeding, and with no previous diagnosis or use of medication for chronic disease. Our study is a secondary analysis of a cross-sectional study, and we accepted the sample size from African-PREDICT. For this study, we included 1153 participants with complete dietary, SES and CV data. All other procedures have been published previously^(30)^.

### Data collection

#### Dietary intake data

Three 24-h dietary recall interviews were conducted with each participant. The first interview took place on the same day as all data collection for the study. Two follow-up interviews were done within a week after the first interview ensuring that a weekend day’s consumption was also included. The fieldworkers were trained to use the five-step multiple-pass approach in conducting the 24-h dietary recall interview^(31)^. Each fieldworker used a standardised dietary collection kit containing pictures as examples, food packages, household measurement tools and food models. Food portion sizes were estimated using plastic food models, household utensils, food packaging materials and a food portion photograph book. The 24-h dietary recalls were coded according to the food composition tables of the South African Medical Research Council (SAMRC)^(32)^; its food quantities manual^(33)^ was then used to convert household measures to grams. If the foods were not available, they were purchased, weighed and recorded for future use. Before analysis, the codes and amounts of each 24-h dietary record were checked against the original 24-h dietary recall to ensure that the data were coded and captured correctly. Nutrient analysis of the baseline dietary data was conducted by the SAMRC at its Biostatistics Unit, using the food composition tables for South Africa^(32)^. The individual average energy and nutrient intake were calculated from the three 24-h recalls for each participant. The EER for each participant was calculated based on established formulas consider factors such as age, sex, weight, height and activity level to estimate the energy needs of each participant^(34)^. The calculated EER for each participant was then compared with their reported energy intake. Participants were categorised based on whether their reported energy intake met or fell below their calculated EER. The percentage of participants with consumption below the estimated average requirements (EAR) or adequate intake was calculated^(34,35)^.

#### Socio-economic status

A questionnaire adapted from Kuppuswamy’s socio-economic scale was used to collect the data. SES of each participant was calculated using a point system that was adapted from Kuppuswamy’s Socioeconomic Status Scale for a South African environment, scoring participants in three categories^(36)^. The scale consisted of three categories: skill level (occupation), education of the head of the household and monthly household income. Each participant’s SES was classified as level 1 (low), level 2 (middle) or level 3 (high) for the three categories combined.

#### Other measurements

Clinic blood pressure measurements were taken at the brachial artery twice on both the left and the right arm (DINAMAP, GE Healthcare), with the participant in a sitting, resting position and the arm at heart level. The mean of the four readings was used for all subsequent analyses.

Anthropometric measurements were performed according to standard methods and included: height (SECA 213 Portable Stadiometer), weight (SECA 813 Electronic Scales) and waist circumference (Lufkin Steel Anthropometric Tape (W606PM)). BMI was calculated using the formula: weight (kg)/height (m)^2(37)^.

Urinary Na and potassium were measured using ion-selective electrode potentiometry on the Cobas Integra 400 plus (Roche) and used as proxies for Na and potassium intake. Participants received instructions on the protocol for 24-h urine collection, starting on the morning of participation, and 24-h urine samples were considered complete if the total urinary volume was ≥ 300 ml^(38)^. Daily Na and potassium excretion was calculated by multiplying urinary Na and potassium concentrations (mmol/l) by the total volume of urine (in litres) and expressed as mmol/d. Salt intake (g/d) was calculated by multiplying the Na (as mmol) by 58·9 (combined molecular weight of Na and chloride)^(39)^.

#### Determination of nutrient patterns

Nutrient patterns were derived from the 24-h dietary recall data using factor analysis with the principal axis factoring extraction method with the correlation matrix to standardise data^(40)^. The factor analysis was conducted on twenty variables, representing the number of nutrients, to develop several nutrient patterns that would explain most of the variances in the observed nutrient intake. The reliability of the factor analysis was verified by using the Kaiser–Meyer–Olkin (KMO > 0·8) test. Factors were rotated by the orthogonal Varimax rotation method to provide a simpler structure and to improve interpretation based on the nutrient pattern’s natural explanation and eigenvalue of > 1·00. The nutrient patterns were named according to the nutrients with the highest loadings in each one of the factors, where the loading represents the correlation between the factor and the input nutrient. To define the extent to which each of the input nutrients contributes to the value of each of the factors, the nutrients with absolute loadings greater than or equal to 0·4 on a given factor were retained for nutritional interpretation^(40)^.

Factor scores for every nutrient pattern were computed for each individual member of the study population to indicate the degree to which each person’s nutrient intake conformed to the identified patterns. For each nutrient pattern, participants were grouped into three categories according to tertiles of factor scores; the distribution of participants across the tertiles of each pattern according to/within the corresponding ethnic group and SES group was subsequently determined.

### Statistical analysis

The Statistical Software Package SPSS, version 27 (IBM) was used for all statistical analyses. Normally distributed data were reported as mean and sd. The dietary data were log-transformed, and after log-transformation, most were still not normally distributed. Dietary data are therefore reported as medians (25th; 75th percentile). Categorical values were expressed as percentages and frequencies. The Mann–Whitney *U* test was used to compare two ethnic groups within the three SES groups, and the Kruskal–Wallis test was used to compare three SES groups within the two ethnicities. Differences between the participants’ proportions across the tertiles of nutrient patterns scores were determined using the Pearson *χ* ^2^ test with adjusted *P*-values (Bonferroni method). A *P*-value < 0·05 was taken as significant.

## Results

### Characteristics of the African-PREDICT study participants

The study group consisted of 1153 healthy participants, of whom 51·0 % were females, 49·7 % were black and 50·3 % were white participants. The former had significantly lower BMI than the latter, but significantly higher systolic blood pressure and diastolic blood pressure (Table 1). Na urinary excretion was higher in the black participants, whereas their potassium urinary excretion was lower than in white participants, and the differences were statistically significant.

**Table 1.** The baseline characteristics of the African-PREDICT study participants

Participant’s characteristics	Total baseline group			Black participants			White participants		
	*n*	Mean	sd	*n*	Mean	sd	*n*	Mean	sd
Age, years	1153	25·5	3·1	573	24	3·0	580	25	3·0
Gender									
Males, %	562	49		286	50		276	48	
Females, %	591	51		287	50		304	52	
Low SES, %	454	39		337	58*		117	20*	
Middle SES, %	336	29		158	28		178	31	
High SES, %	363	32		78	14*		285	49*	
BMI, kg/m^2^	1153	25	5·5	573	24·6	5·6*	580	25·5	5·4*
WC, cm	1153	80·2	12·5	573	77·9	10·8*	580	82·4	13·7*
SBP, mmHg	1153	118·6	11·7	573	119·5	11·6*	580	117·7	11·7*
DBP, mmHg	1153	78·6	7·7	573	79·7	8·0*	580	77·4	7·2*
24-h Na urinary excretion, mmol/d	971	156·5	103·3	444	159·1	95·9*	527	154·2	109·3*
24-h potassium urinary excretion, mmol/d	1008	50·7	33·6	466	41·9	28·5*	542	58·1	35·7*

SES, socio-economic status; WC, waist circumference; SBP, systolic blood pressure; DBP, diastolic blood pressure.

Data are reported as mean (sd), unless otherwise indicated.

* *P* < 0·05.

### The energy and nutrient intake

The nutrient intake of the total group of participants, and stratified by ethnicity, is reported in online supplementary material, Supplemental Table S1. More than 70 % of total participants did not meet the EER, and there was no significant difference in the percentage between ethnic groups, although the median daily energy intakes in the black participants were less than in White participants, 7460 kJ and 8046 kJ, respectively.

In terms of macronutrient distribution, the protein, fat and carbohydrate intake of both the black and White participants, as well as the total group, fell within the Acceptable Macronutrient Distribution Ranges (AMDR). While there was no statistically significant difference in the percentage of total energy (TE) derived from protein intake between the ethnic groups, the black participants showed a lower contribution of energy from animal protein and a higher contribution from plant protein. The contribution of fat intake to TE was lower in the black participants (31·6 %) than in the white participants (38·5 %), and the opposite was for carbohydrate intake (56·7 % for black and 45·7 % for white), respectively.

There were significant differences in the intake of most micronutrients between ethnic groups (see online supplementary material, Supplemental Table S1). Black participants had a lower intake of most micronutrients, except for fibre, Fe, Zn, thiamine and niacin, than white participants. Fibre intake was lower than the recommendations for all participants, and there was no difference in intake between ethnicities. A higher added sugar consumptions was reported by the white participants than by the black participants.

The macro- and micronutrient intake, stratified by SES groups, is reported for black and white participants in Table 2. The highest percentage of participants with TE below the EER was observed in the high SES group among black participants and in the low SES group among white participants. The percentages of TE from total protein were similar across all SES and ethnic groups, ranging from 14·5 % to 17·0 %, with a slightly higher contribution in white participants.

**Table 2.** Nutrient intakes of the black and white participants within the socio-economic groups

	Low SES (*n* 454)				Middle SES (*n* 336)				High SES (*n* 363)			
	Black (*n* 337)		White (*n* 117)		Black (*n* 158)		White (*n* 178)		Black (*n* 78)		White (*n* 285)	
	Median P25–P75		Median P25–P75		Median P25–P75		Median P25–P75		Median P25–P75		Median P25–P75	
Energy and nutrients intake per d												
Total energy (TE), kJ*,^†^	7974^a^	5794–9216	7299^a^	5623–9343	7749^a^	6079–10 241	8195^b^	6553–10 438	7090^b,e^	5869–9030	8286^b,d^	6670–10 001
Total protein, g	62·8^a^	45·9–80·3	71·5^a^	52·8–90·3	63·4^a^	48·6–83·0	79·2^b^	60·2–103·4	59·8^b, e^	47·2–85·7	78·2^b, d^	59–100·7
Animal protein, g	29·2	20·0–45·0	48·7	28·5–64·2	34·5	20·3–50·6	51·2	35·8–71·9	35·6	22·2–51·3	51·0	37·5–72·1
Plant protein, g	22·8	16·0–28·7	16·4	12·1–24·3	21·7	15·3–30·1	27·4	12·2–23·6	18·9	13·8–25·5	16·4	12·0–21·6
Total CHO, g	239·7^a^	183·3–309·3	198·7^a^	147·7–255·3	249·1^b^	183·0–353·3	208·3^b^	157·7–268·7	216·6^a^	170·1–301	206·2	166·9–266·7
Total fat, g	57·4^a^	41·9–78·0	70·1^a,e^	47·1–94·8	59·9^b^	39·1–82·6	83·5^b, e^	63·9–111·7	58·9^c^	45·9–84·4	81·6^c,e^	57·5–105·4
Saturated fat, g	17·1^a^	11·6–23·7	22·3^a,e^	15·2–30·9	16·8^b^	11·0–24·5	29·2^b, e^	20·6–38·5	18·8^c^	13·9–24·9	26·6^c,e^	19·5–35·6
MUFA, g	19·4^a^	14–26·5	25·0^a^	16·3–34·1	19·3^b^	12·8–28·1	28·8^b^	21·6–40·5	20·5	15·6–29·2^c^	28·2^c^	20–36·7
PUFA, g	13·6	9·1–20·0	13·9	8·2–21·1	13·6	8·6–20·5^b^	16·0^b^	10·3–25·3	12·5	8·9–18·9^c^	16·1^c^	10·6–23·7
Total fibre, g	13·2	9·0–17·6	12·9	8·5–16·5	14·7	9·7–19·2	12·3	9·0–19·1	13·1	9·6–19·2	13·1	9·8–18·4
Ca, mg	271·3^a^	155·4–451·7	490·1^a,e^	271·5–770·9	274·9^b^	162·6–440·5	665·6^b, e^	422·1–924·8	330·1^c^	212·6–543·1	639·9^c,e^	441·1–890·4
Total Fe, mg	12·5	9·2–16·6	11·5	9·1–14·9	11·8	8·6–16·4	12·2	9·1–17·5	11·5	9·2–16·1	12·1	9·3–16·9
Mg, mg	180·6^a^	140·6–240·1	214·5^a,e^	160·5–265·5	192·2^b^	142·8–251·8	227·0^b, e^	181·8–313·5	183·6^c^	139·4–228·6	239·0^c,e^	193·1–305·3
Phosphorus, mg	751·1^a^	559·5–978·1	995·5^a,e^	729·5–293·2	800·2^b^	596·8–1068·8	1134^b, e^	856·1–1491·8	769·5^c^	578·8–997·5	1143·2^c,e^	916·2–1429·2
Potassium, mg	1420^a,d^	1074–1895	2099^a,e^	1446–2563	1560^b,d^	1188–2247	2272^b,e^	1809–2818	1663^c,d^	1287–2184	2364^c,e^	1893–2894
Zn, mg	10·5	7·6–13·6	10·3	7·5–13·5	10·1	7·6–14·4	11·0	8·6–14·3	9·3	7·2–12·8	10·3	8·1–13·9
Copper, mg	0·76^a,d^	0·60–1·0	0·86^a,e^	0·68–1·2	0·85^b, d^	0·63–1·1	1·0^b, e^	0·86–1·4	0·88^c, d^	0·68–1·1	1·0^c,e^	0·8–1·3
Vitamin A (RE), µg	475·0	326·4–706·3	564·7	392·2–1067	569·5	355·2–921·2	596·6	388·4–956·4	549·4	323·4–1031·1	626·6	412·1–949·6
Thiamine, mg	1·3	0·94–1·7	1·2	0·93–1·6	1·4	0·93–1·8	1·3	1·0–1·7	1·2	0·85–1·6	1·3	1–1·7
Riboflavin, mg	1·3^a^	0·80–2·0	1·5^a,e^	1·0–2·1	1·2	0·76– 2·0^b^	1·8	1·2–2·5^b, e^	1·3	0·96–2·1^c^	1·8^c,e^	1·3–2·4
Niacin, mg	24·6	18·0–31·7	20·8	15·4–28·8	23·5	18·2–31·2	23·1	17·5–30·6	22·4	17–30·5	23·4	17·9–30·1
Vitamin B_6_, mg	3·4^a,d^	2·2–4·6	2·4^a^	1·6–3·5	2·8	2·0–4·3^b, d^	2·2	1·5–3·3^b^	2·6	1·6–3·3^d^	2·3	1·6–3·1
Folate, µg	268·0^a,d^	187·2–353·1	189·4^a^	133·1–298·7	266·0^b,d^	180·7–408·0	217·5	137·1–298^b^	210·8^c, d^	141·1–319·1	179·9^c^	126·9–249·4
Vitamin B_12_, µg	2·3^a^	1·2–4·0	3·3^a,e^	2·2–5·2	2·7	1–4·6^b^	4·0^b, e^	2·8–5·9	2·8^c^	1·6–4·7	4·0^c,e^	2·6–6·3
Pantothenic acid, mg	4·4	2·9–6·6	4·5	2·9–6·3	4·7	3·1–7·2^b^	5·2	3·7–7·3^b^	4·3	3–6·2^c^	5·5	3·8–7·8^c^
Biotin, µg	18·8	13·3–27·1	23·4^e^	14·1–29·6	19·9	13·1–28·9^b^	26·7	19·5–37^b, e^	18·4	13·4–23·8^c^	25·3	18·6–34·6^c,e^
Vitamin C, mg	16·5^a, d^	7·1–31·9	30·2^a,e^	13·4–72·2	24·9	11·3–52^b, d^	41·5	20·6–118^b, e^	35·7	17·4–82·8^c, d^	55·7	27–100·2^c,e^
Vitamin D, µg	2·4	1·0–5·7	2·6	1·2–5·5	2·1	0·81–4·4^b^	3·2	1·6–5·4^b^	2·1	1·1–3·6^c^	3·1	1·8–5·5^c^
Vitamin E, mg	8·0^d^	4·7–12·5	8·5	4·7–13	6·9	4·3–11·4^b, d^	9·5	5·9–14·8^b^	6·0	4·3–9·2^c, d^	8·6	5·7–13^c^
Pantothenic acid, mg	4·4	2·9–6·6	4·5	2·9–6·3	4·7	3·1–7·2^b^	5·2	3·7–7·3^b^	4·3	3–6·2^c^	5·5	3·8–7·8^c^
Energy distribution	Mean	sd	Mean	sd	Mean	sd	Mean	sd	Mean	sd	Mean	sd
Total protein, % TE^‡^	14·5	4·1	16·8	4·5	14·5	3·8	16·9	4·7	14·7	4·3	17·0	5·3
Total CHO, % TE	55·5	9·3	46·5	12·7	56·2	10·3	43·7	10·2	53·3	9·9	44·7	10·5
Sugar, % TE	11·4^d^	8·3	11·5^e^	7·0	12·2^d^	7·6	12·0^e^	8·2	14·1^d^	7·1	14·4^e^	8·2
Added sugar, % TE	4·1^a^	6·0	6·9^a,e^	7·6	4·7^b^	7·2	7·3^b,e^	7·0	4·2^c^	4·8	5·4^c,e^	5·6
Total fat, % TE	30·2	8·4	35·9	10·6	29·8	9·1	38·8	8·7	32·1	9·3	37·3	8·6
Saturated fat, % TE	8·9	3·1	11·8	3·9	8·6	3·3	13·1	3·7	9·0	3·1	9·9	3·1
MUFA, % TE	10·2	3·3	12·7	4·9	10·2	3·8	13·5	3·8	11·3	3·8	12·8	3·8
PUFA, % TE	7·6	3·7	7·4	4·2	7·6	4·5	8·0	3·9	7·4	3·2	7·8	3·4
Percentage of study participants below the dietary reference intakes§												
Total energy < EER, %	71·2		82·8		64·1		72·6		79·2		69·8	
Total protein < EAR, %	32·9		22·4		28·2		11·2		26·0		12·3	
Ca < EAR^4^, %	93·2		77·8		92·4		66·9		96·2		67·4	
Total Fe < EAR, %	12·8		16·2		15·8		10·0		14·1		13·0	
Mg < EAR, %	86·9		82·1		82·9		72·5		87·2		70·5	
Phosphorus < EAR, %	29·1		12·8		23·4		5·1		25·6		3·2	
Potassium < EAR, %	92·7		83·2		92·7		75·4		85·9		75·2	
Zn < EAR, %	29·7		26·5		26·0		18·0		30·8		21·4	
Copper < EAR, %	100·0		100·0		100·0		100·0		100·0		100·0	
Vitamin A < EAR, %	61·1		47·9		47·5		43·8		50·0		42·1	
Thiamine < EAR, %	25·8		27·4		23·4		23·6		33·3		25·3	
Riboflavin < EAR, %	34·4		23·9		36·7		16·3		24·4		13·3	
Niacin < EAR, %	5·9		3·4		3·8		7·3		5·1		4·9	
Vitamin B6 < EAR, %	5·3		10·3		6·3		10·7		11·5		11·9	
Folate < EAR, %	54·9		47·0		50·0		42·1		69·2		69·5	
Vitamin B12 < EAR, %	42·4		19·7		41·1		11·2		32·1		14·0	
Pantothenic acid < EAR, %	58·2		59·0		53·2		47·2		60·3		39·3	
Biotin < EAR, %	78·9		76·1		77·9		61·2		85·9		64·9	
Vitamin C < EAR, %	89·3		72·7		81·7		59·6		68·0		58·3	
Vitamin D < EAR, %	90·2		94·0		94·3		92·1		96·2		92·3	
Vitamin E < EAR, %	73·3		70·1		76·6		66·3		88·5		68·1	

SES, socio-economic status; CHO, carbohydrate; RE, retinol equivalent; EER, Estimated Energy Requirements; EAR, estimated average requirements.

* Reported as median (25th–75th percentile), all such values.

† Kruskal–Wallis test used for non-parametric variables to compare SES groups according to the ethnicity; Mann–Whitney *U* test used for non-parametric variables to compare nutrient intake between two ethnic groups within the SES; ^a,b,c^, the values with different letters in superscript differed significantly,^e,d^, the values with different letters in superscript differed significantly.

‡ Reported as mean (sd), all such values.

§ Reported as categorical data, all such values.

The percentages of participants with protein intake below the EAR varied across groups, with the highest percentages of black participants in the low SES group and the lowest in the white middle SES group. The highest intake of animal protein was observed in the white middle and high SES groups, while the lowest was observed in the black low SES group. The highest intake of plant protein was observed in the black low and white middle SES groups, while the lowest intake was observed in the white low and high SES groups.

The highest intake of total carbohydrates was observed in the black low and middle SES groups. The percentages of TE from added sugar were generally higher in the white participants compared to the black participants.

Both black and white participants did not meet the EAR for seventeen of the nineteen reported micronutrients, and consistently across the SES groups, more than 50 % of participants from each ethnic group did not meet the EAR for several nutrients, including Ca, Mg, folate, pantothenic acid and biotin, as well as vitamins A, C, D and E. Also, consistently across SES groups, black participants reported a significantly lower intake of protein, total fat, Ca, Mg, potassium and phosphorus, as well as vitamins A, B_12_, C and E, compared to white participants (all *P* < 0·05). However, for micronutrients such as niacin, thiamine, Fe, Zn and fibre, there were no significant differences between the two ethnic groups stratified according to SES. Although white participants generally reported the higher intake for most nutrients, black participants in the high SES group also had high intake. However, for some nutrients, the highest intake was observed in the middle SES groups for both ethnicities.

### Nutrient patterns identified by factor analysis

Exploratory factor analysis was conducted and confirmed that the multivariate reduction technique applies to our study sample (KMO > 0·8). Considering the variation in dietary energy intake by participants, the amounts of individual nutrient intakes were adjusted for energy. The nutrient pattern matrix and names assigned are presented in online supplementary material, Supplemental Table S2. Four factors – corresponding to four nutrient patterns – were extracted, which explained 51·6 % of the variance of nutrient intake. The first nutrient pattern (factor 1) is mainly representative of animal protein and saturated fat; the second (factor 2) represents Mg, potassium, various micronutrients and fibre; the third (factor 3) is mainly representative of plant protein, B-vitamins and minerals; and the fourth nutrient pattern (factor 4) mainly reflects vitamin E and PUFA. Each factor contributes a percentage to the total variance explained, namely 15·5 %, 14·6 %, 13·7 % and 7·8 %, respectively (see online supplementary material, Supplemental Table S2). Factors are named according to the nutrients with relatively high loadings (as dominant components) that cluster around the same pattern^(40)^. Thus, factor 1 was named ‘Animal protein and saturated fat’ nutrient pattern; factor 2 is ‘Magnesium, potassium, calcium, phosphorus, and fiber’ nutrient pattern; factor 3 is ‘Plant protein, B–vitamins, zinc, and iron’ nutrient pattern; and factor 4 is ‘Vitamin E and PUFA’ nutrient pattern. A factor score for each nutrient pattern was assigned to each participant.

The distribution of participants according to ethnic group across the tertiles (T1–T3) of each nutrient pattern score is shown in Table 3. A significantly higher proportion of black participants fell within the T1, and a lower proportion in the T3 of the first and second nutrient patterns, compared to white participants (*P* < 0·001). In contrast, within the third nutrient pattern, a significantly lower proportion of black participants fell within T1 and a higher proportion in T3, than for white participants (*P* = 0·005). There was no significant difference between the corresponding proportions for black and white participants regarding the fourth nutrient pattern.

**Table 3.** Comparisons of participants’ proportions by tertiles of nutrient pattern scores

Tertiles of nutrient patterns scores	Black participants	White participants	*P* value
‘Animal protein and saturated fat’ nutrient pattern			
T1	75·6^a^	24·4^b^	<0·001
T2	55·2^a^	44·8^b^	
T3	19·7^a^	80·3^b^	
‘Magnesium, potassium, Ca, phosphorus and fibre’ nutrient pattern			
T1	74·6^a^	25·4^b^	<0·001
T2	51·2^a^	48·8^a^	
T3	24·9^a^	75·1^b^	
‘Plant protein, B-vitamins, zinc, and iron’ nutrient pattern			
T1	35·4^a^	64·3^b^	0·005
T2	51·4^a^	48·6^a^	
T3	63·8^a^	36·2^b^	
‘Vitamin E and PUFA’ nutrient pattern			
T1	52·8^a^	47·2^a^	0·451
T2	48·8^a^	51·2^a^	
T3	49·0^a^	51·0^a^	

T, tertile. Values are expressed as percentage of the subgroups for categorical variables. T1 *n* 385, T2 *n* 384, T2 *n* 384.

*χ* ^2^ test used for categorical variables; *z*-test with adjusted *P* values (Bonferroni method). Superscript letters in a row that are the same indicate a subset of subgroups that did not differ significantly from each other; superscript letters in a row that differ denote a subset of subgroups that differ significantly from each other at the *P* < 0·05.

The distribution of participants according to their SES in terms of T1–T3 of each nutrient pattern score is shown in Table 4. A significantly higher proportion of participants with low SES fell within T1 and a smaller proportion was in T3 compared to those with high SES for the first nutrient pattern (53·1 % and 18·6 %, 27·1 % and 42·0 %) and for the second nutrient pattern (54·8 % and 16·6 %, 22·9 % and 47·4 %), respectively (all *P* < 0·001).

**Table 4.** Comparison of participant’s proportions in three socio-economic groups by tertiles of nutrient pattern scores

Tertiles of nutrient patterns scores	Low SES	Middle SES	High SES	*P* value
‘Animal protein and saturated fat’ nutrient pattern				
T1	53·1^a^	28·4^b^	18·6^c^	<0·001
T2	38·4^a^	30·1^a^	31·5^a^	
T3	27·1^a^	30·9^b^	42·0^c^	
‘Magnesium, potassium, Ca, phosphorus, and fibre’ nutrient pattern				
T1	54·8^a^	28·6^b^	16·6^c^	<0·001
T2	38·3^a^	31·2^a^	30·5^a^	
T3	22·9^a^	29·7^b^	47·4^c^	
‘Plant protein, B-vitamins, zinc, and iron’ nutrient pattern				
T1	27·8^a^	31·9^b^	40·4^b^	<0·001
T2	41·3^a^	28·2^a^	30·5^a^	
T3	49·3^a^	27·3^b^	23·3^b^	
‘Vitamin E and PUFA’ nutrient pattern				
T1	38·3^a^	29·9^a^	31·8^a^	0·362
T2	36·5^a^	29·7^a^	33·9^a^	
T3	43·3^a^	28·7^a^	28·9^a^	

T, tertile. Values are expressed as percentage of the subgroups (socio-economic groups) for categorical variables: T1 *n* 385, T2 *n* 384, T2 *n* 384.

*χ* ^2^ test used for categorical variables; *z*-test with adjusted *P* values (Bonferroni method).

Superscript letters in a row that are the same denote a subset of subgroups that did not differ significantly from each other; superscript letters in a row that differ indicate a subset of subgroups that differ significantly from each other at the *P* < 0·05 significance level.

In contrast, for the third nutrient pattern, a significantly lower proportion of participants with low SES fell within the T1 subgroup; a higher proportion was in T3 compared to high SES (27·8 % and 40·4 %, and 49·3 % and 23·3 %) (*P* < 0·001). There was no significant difference between the proportions of SES groups regarding the fourth nutrient pattern.

## Discussion

The analysis of the baseline dietary intake of young adults participating in the African-PREDICT study aimed to analyse and compare the energy and nutrient intake of black and white participants. The results show significant differences between the two ethnic groups, as well as across the three SES categories. Moreover, our findings reveal that more than two-thirds of the participants fell below the recommended levels of EER, suggesting potential issues of insufficient energy intake in this population. However, this reported energy under-intake appears inconsistent with the observed BMI, which suggests adequate energy intake to maintain healthy body weight. It is important to acknowledge that dietary data are prone to misreporting, which could explain the discrepancies between reported energy intake and actual EER. This aligns with previous research showing substantial under-reporting, up to 83 %, among South African participants, particularly in those with higher BMI^(41)^. Still, comparing our results with other studies is challenging due to the scarcity of studies reporting the proportions of the South African population with energy intake below the recommended requirements. Nevertheless, studies that report only means and medians of energy intake consistently indicate that it falls below the recommended levels, though there were some increases in energy intake observed in 2010 for the PURE-NWP-SA participants^(11,19)^. Under-reporting of energy intake likely extends to other nutrients, leading to widespread underestimation. Since dietary assessments rely on self-reported data, similar biases may affect the reporting of macronutrient and micronutrient intake^(42)^.

In terms of macronutrient distribution as a percentage of TE, both ethnic groups in our study met the AMDR for protein, fat and carbohydrates, indicating their intake was within the recommended levels. Yet, the proportion of energy derived from total protein and carbohydrates was closer to the lower end of the AMDR range in both ethnic groups. The observation that stands out is the higher contribution of energy from animal protein compared to plant protein, regardless of ethnicity. In fact, the amount of energy derived from animal protein was more than double that from plant protein. This indicates a preference for animal-based food sources in the diet of both ethnic groups. Although, notable differences were observed in our study in the proportion of macronutrients contributing to TE between the two groups, white participants had a higher percentage of TE derived from animal protein and fat, including saturated fats. In contrast, black participants had a higher percentage of TE-derived from carbohydrates and plant proteins, indicating a high consumption of carbohydrates-rich food like grains and starchy vegetables, and plant protein intake in the form of beans and other legumes. Additionally, white participants reported higher consumption of added sugar compared to black participants, although intake was still lower than the WHO’s recommended 10 % of total energy intake^(43)^. Furthermore, a further reduction to below 5 % of total energy intake is suggested for additional health benefits. This recommendation is based on the potential negative health impacts of excessive added sugar consumption, particularly about obesity and certain non-communicable diseases. It is important to note that fibre intake was below the recommended levels for all study participants, regardless of ethnicity. This suggests that foods rich in dietary fibre, which are predominantly fruit and vegetables, beans and other legumes, and whole grains, are not being consumed in sufficient quantities. These trends in macronutrient distribution, with a few exceptions, include increased energy intake from animal protein and saturated fat, and decreased energy from plant protein and carbohydrates, despite an increase in added sugar consumption, were also observed in the PURE-NWP-SA study^(19)^.

Our findings demonstrate a substantial prevalence of insufficient micronutrient intake among the study participants: over 50 % of all participants, regardless of their ethnicity, did not meet the EAR or adequate intake for several essential micronutrients. These included potassium, Mg, folate, Ca, biotin and vitamins A, C, D and E. These deficiencies are concerning as the nutrients, typically obtained through a diverse and nutrient-rich diet, are crucial for cardiovascular health by regulating BP and heart function, and by reducing inflammation and oxidative stress, the key factors in preventing CVD and avoiding hypertension (HT)^(44)^. Furthermore, despite the mandatory fortification of maize meal and wheat flour in South Africa since 2003, our findings indicate that a significant proportion of individuals still fall below the recommended intake levels for certain fortified micronutrients, such as vitamin A and folic acid, contributing to a diet of poor quality.

The lack of common reporting on participants with intakes below the EAR in published studies hinders the comparison of results and a comprehensive understanding of dietary adequacy in South Africa. However, the use of EAR in assessing nutrient adequacy is crucial in identifying vulnerable populations and guiding interventions to address nutrient deficiencies. It also enables monitoring of nutrient intake changes over time and evaluating the effectiveness of interventions in improving dietary patterns. To overcome the lack of specific studies, we can refer to a review conducted by Mchiza *et al.* ^(11)^, which highlights low nutrient intakes among South Africans falling below recommended levels.

Our study findings are consistent with results from studies indicating disparities in dietary intake across socio-economic and ethnic groups. Studies conducted worldwide consistently show that individuals from socio-economically disadvantaged groups, particularly those within lower SES groups, tend to exhibit unhealthy dietary habits characterised by the consumption of energy-dense foods with low nutrient density^(45–47)^.

The differences in nutrient intake between ethnic groups were consistently observed in our study, with black participants reporting lower intakes of essential micronutrients compared to white participants, regardless of SES. This aligns with previous research indicating that black participants tend to have a less diverse and more monotonous diet, which contributes to lower nutrient intake^(15,17,48)^. A longitudinal study, the PURE-NWP-SA, further supports these findings, revealing a high prevalence of inadequate intakes of Ca and potassium among black participants across all study groups, with minimal improvement over time during the study period from 2005^(19)^. In fact, up to 80 % of the black participants did not meet the recommended nutrient intake levels for most nutrients^(18,19)^. Moreover, inadequate intakes of Ca and potassium were highly prevalent among the PURE-NWP-SA participants from all study groups, with minimal improvement observed over the study period from 2005 to 2010. These observations can be attributed to the consumption of energy-dense but nutrient-poor foods^(17)^.

The results of nutrient pattern analysis revealed four distinct nutrient patterns among study groups that represent different dietary habits and food choices among South African adults and are associated with the different ethnic groups and SES categories.

The adherence to the nutrient pattern characterised by a high intake of animal protein, saturated fat and biotin, along with the pattern featuring high intake of Mg, potassium, Ca, phosphorus and fibre, was more prevalent among white participants and those with high SES in our study. This suggests that these dietary habits are associated with ethnic groups and SES in South African young adults. The higher adherence of white participants to the first nutrient pattern is noteworthy, as it aligns with their significantly higher BMI and waist circumference compared to the black group. This observation is in line with previous research indicating that this type of nutrient pattern may increase the risk of obesity, CVD, diabetes and certain cancers^(49)^. The shift towards increased animal protein consumption in black participants, as observed in the PURE-NWP-SA study, further raises concerns about the potential negative health effects of these dietary patterns.

Regarding the poor adherence of black participants to the second nutrient pattern, our results are consistent with lower urinary potassium excretion of 41·9 mmol/d in this group. Furthermore, the total baseline group showed a potassium urinary excretion of 50·7 mmol/d, which is notably lower than the WHO-recommended minimum of 90 mmol/d^(50)^. Additionally, the results showed higher urinary Na levels in black participants, along with significantly higher BP levels. These observations align with the widely recognised importance of consuming adequate amounts of essential nutrients such as Mg, potassium, fibre, Ca and healthy protein while limiting fats and Na, as this is associated with improved overall health and a reduced risk of CVD^(51,52)^. Furthermore, adopting plant-based dietary patterns has demonstrated protective effects on cardiovascular function by enhancing endothelial function, regulating vasodilation, reducing oxidative stress, mitigating inflammation responses and modulating BP^(4–8,53)^. However, while plant-based foods are rich in plant proteins and help to reduce saturated fat consumption compared to animal-based foods, they can also be lower in some essential amino acids, which need to be supplemented by other foods. Our findings underscore the significance of advocating for the SAFBDG, which recommends adopting a diverse and nutrient-rich diet that includes various protein sources as a strategic approach to improve overall health status^(22)^.

Our analysis revealed that over 60 % of black participants adhere to the third nutrient pattern, characterised by a high intake of micronutrients mainly obtainable from cooked maize porridge and bread. This suggests a dietary restriction among black South African adults, with maize meal and bread being the predominant staple foods^(15)^. To address the micronutrient deficiencies, the South African Department of Health initiated mandatory fortification of staples (bread flour and maize meal) with essential vitamins (thiamine, riboflavin, niacin, pyridoxine, folic acid and vitamin A) and minerals (Fe and Zn) in 2003^(21)^.

The consumption of fortified staple foods has the potential to significantly improve nutrient intake, explaining the relatively low percentage of our study population not meeting EAR for some nutrients, like Fe, pyridoxine and niacin. However, as previously indicated, deficiencies in some of the fortified nutrients persist among black participants, with more than a quarter or even a third failing to meet recommended intake levels.

Available dietary data from various studies consistently demonstrate that nutrient intake among different South African study groups falls mostly below recommended requirements, particularly for black participants^(11)^. Samples of maize meal and white bread flour analysed in 2012 were found to be insufficiently fortified, which could partially explain our participants’ relatively low intake of these nutrients^(54)^. However, it is important to note that our study used food composition tables, which assume 100 % fortification values for bread and maize consumed. This means that the observed nutrient intake below EAR in our study cannot be directly attributed to inadequate fortification based on our data alone. Thus, the effects of staple fortification on South African adults’ micronutrient status remain uncertain^(11)^.

Moreover, overconsumption of fortified staple foods, along with dietary fats and added sugars, may increase health risks for South Africans. To mitigate these risks, the SAFBDG recommends replacing processed and refined carbs with non-starchy vegetables, healthy fats, whole fruits, legumes and whole grains, which will enhance dietary diversity and ensure adequate micronutrient intake^(22)^. However, the overall observed results regarding nutrient intake in our study suggest that participants may not be receiving sufficient health protection from their diets, which could potentially contribute to the increased prevalence of cardiovascular issues among the study population. PURE-NWP-SA also highlighted a significant increase in the consumption of sugar-rich foods and a lack of essential nutrients among study participants from 2005 to 2010^(19)^, with 24 % of those with normal BP in 2005 developing HT by 2010^(55)^. Thus, our findings underscore nutritional issues that may contribute to an increased risk of cardiovascular problems in the African-PREDICT cohort.

### Strengths and limitations

Our study provides valuable insights into nutrient patterns among young adults in a specific province of South Africa, serving as a reference for future longitudinal studies on dietary intake and health outcomes. By focusing on this study population, we address a gap in existing research, which has often overlooked young adults. The identification of nutrient patterns and the exploration of cumulative effects of various nutrient combinations offer promise in planning effective intervention studies and understanding the role of diet in the onset of cardiovascular issues. However, it is important to acknowledge that the generalisability of our findings may be limited by several factors inherent to the study design and sample characteristics. African-PREDICT participants were not randomly selected from the entire South African population but were recruited from a specific province. This non-random selection process may limit the generalisability of our results to other regions or provinces within South Africa. The study focused on young adults aged 20–30 years, which may further restrict the applicability of our findings to other age groups within the population. Different age groups may exhibit varying dietary habits and nutrient intake patterns, thus limiting the generalisability of our results to older or younger populations. In addition, the study only included black and white participants, representing a bi-ethnic sample, which may not fully capture the diversity of South Africa’s various ethnic groups, each with distinct cultural and dietary practices. Therefore, our findings may not generalise to other ethnic groups.

An important limitation is the potential under-reporting of dietary intake, common in self-reported data. The inconsistency between reported energy intake and observed BMI highlights this issue and suggests that under-reporting may also affect the estimates of other nutrients. Nutrient values in any food composition database reflect averages and therefore gives an approximate indication of the nutrient content of a food, further compounding this issue. Such biases should be considered when interpreting the study’s findings. By acknowledging these limitations and considering suggestions for future research, it can work towards enhancing the generalisability of our findings to inform public health policies and interventions effectively.

### Conclusion

Participants consistently reported lower intakes of essential micronutrients, indicating a high risk of nutrient deficiencies that could potentially contribute to the development of cardiovascular issues. This disparity may be attributed to dietary patterns and food choices prevalent among socio-economically disadvantaged groups, which often consist of energy-dense but nutrient-poor foods. Our findings highlight the need for targeted interventions to improve dietary quality, reduce consumption of unhealthy foods and establish a direct link between nutrient intake and cardiovascular health in the study population. However, there is a clear need for government interventions and strategies to improve nutrient intake and promote healthier dietary intake considering the socio-economic factors influencing food choices.

## Supporting information

Visser et al. supplementary material Visser et al. supplementary material

## Supplementary material

For supplementary material accompanying this paper visit https://doi.org/10.1017/S1368980024002076
