# Supplementary material for: Nutrient intake of young South African adults from the baseline of the African-PREDICT cohort study
Source: Public Health Nutr. 2024 Oct 29;27(1):e234. doi: 10.1017/S1368980024002076 (PMC11705013; doi:10.1017/S1368980024002076)
Supplement: Visser et al. supplementary material [file S1368980024002076sup001.doc]

**Supplementary materials**

**Table S1.** Total median (25^th^–75^th^) nutrient intake for the White and Black participants.

| **Nutrient** | **Total group (n = 1153)** | **Black participants (n = 573)** | | **White participants (n = 580)** |
| --- | --- | --- | --- | --- |
| Total energy, kJ/d | 7748 (6143-9754) | 7460.6 (5870.5–9571) ^a^ | | 8046.6 (6444.8–9945.5) ^a^ |
| Total energy < EER, % | 71.8 | 70.3 | | 73.3 |
| Total protein, g/d | 69.7 (51.8-91.2) | 62.1 (46.3–81.1) ^a^ | | 76.8 (58.3–99.0) ^a^ |
| Total protein < EAR, % | 22.3 | 30.7 | | 14.0 |
| Plant protein, g/d | 18.9 (13.9-25.7) | 21.9 (15.6–28.6) | | 16.7 (12.0–22.5) |
| Animal protein, g/d | 41.0 (25.7-60.0) | 31.6 (20.3–47.4) | | 50.3 (35.5–71.0) |
| Total fat, g/d | 67.6 (48.6-94.2) | 57.9 (41.9–78.6) ^a^ | | 80.9 (56.4–105) ^a^ |
| Saturated fat, g/d | 20.9 (14.5-30.7) | 17.3 (11.7–24.1) ^a^ | | 26.4 (18.6–36.1) ^a^ |
| MUFAs, g/d | 23.0 (16.1-33.2) | 19.7 (13.7–27.0) ^a^ | | 27.8 (1.4–36.9) ^a^ |
| PUFAs, g/d | 14.7 (9.4-22.7) | 13.3 (8.9–20.2) ^a^ | | 15.6 (10.0–24.0) ^a^ |
| Total carbohydrates, g/d | 224.9 (169.9-289.6) | 239.7 (181.4–320.8) ^a^ | | 206.4 (157.5–262.6) ^a^ |
| Total sugar, g/d | 51.8 (28.1-80.8) | 47.7 (26.4–70.4) | | 56.7 (31.2–86.4) |
| Added sugar, g/d | 13.1 (2.1-37.4) | 7.5 (0.0–26.8) ^a^ | | 19.3 (6.8; 45.6) ^a^ |
| Total fibre, g/d | 13.2 (9.3-18.5) | 13.4 (9.3–18.7) | | 12.8 (9.2–18.2) |
| Calcium, mg/d | 424.5 (233.2-706.6) | 280.2 (160.6–464.5) ^a^ | | 616.9 (400.4–879.5) ^a^ |
| Total Iron, mg/d | 12.0 (9.2-16.6) | 12.2 (9.1–16.6) | | 12 (9.3–16.8) |
| Magnesium, mg/d | 208.4 (156.7-274.5) | 182.9 (140.9–243.8) ^a^ | | 231.1 (184.1–298.7) ^a^ |
| Phosphorus, mg/d | 935.5 (686.0-1237.2) | 771.5 (567.4–1015.4) ^a^ | | 1099.2 (846.2–1394.3) ^a^ |
| Potassium, mg/d | 1885 (1341-2536) | 1497 (1142.5–2002.6) ^a^ | | 2272.1 (1758.4–2799.1) ^a^ |
| Zinc, mg/d | 10.4 (7.8-13.9) | 10.3 (7.5–13.7) | | 10.6 (8.1–13.9) |
| Vitamin A (RE), µg/d | 560.4 (359.1-905.1) | 511.7 (328.8–817.5) ^a^ | | 600.3 (394–965.4) ^a^ |
| Thiamine, mg/d | 1.3 (0.9-1.7) | 1.3 (0.9–1.7) | | 1.3 (0.9–1.7) |
| Riboflavin, mg/d | 1.5 (1.0-2.2) | 1.3 (0.8–2.0) ^a^ | | 1.7 (1.2–2.3) ^a^ |
| Niacin, mg/d | 23.4 (17.5-30.1) | 23.9 (17.9–31.4) | | 23 (17–29.9) |
| Vitamin B6, mg/d | 2.6 (1.8-3.8) | 3 (2.0–4.4) ^a^ | | 2.3 (1.6–3.2) ^a^ |
| Folate, µg/d | 173.1 (85.6-284.2) | 259.3 (174–355.2) ^a^ | | 186.0 (129.4–263.5) ^a^ |
| Vitamin B12, µg/d | 3.2 (1.8-5.2) | 2.4 (1.2–4.2) ^a^ | | 3.9 (2.5–6) ^a^ |
| Pantothenic acid, mg/d | 4.9 (3.3-7.1) | 4.5 (2.9–6.8) ^a^ | | 5.2 (3.7–7.3) ^a^ |
| Biotin, µg/d | 22.2 (15.1-31.2) | 19.0 (13.3–27.6) ^a^ | | 25.2 (18.5–33.7) ^a^ |
| Vitamin C, mg/d | 30.4 (14.2-70.9) | 20.2 (9–44.2) ^a^ | | 44.8 (21.7–96.3) ^a^ |
| Vitamin D, µg/d | 2.6 (1.2-5.3) | 2.2 (1–4.8) ^a^ | | 3.1 (1.6–5.5) ^a^ |
| Vitamin E, mg/d | 8.2 (4.9-12.7) | 7.4 (4.5–11.8) ^a^ | | 8.8 (5.7–13.4) ^a^ |
| **Energy distribution** | |  |  |  |
| % of TE from protein | 15.3 (5.4-27.6) | 14.0 (11.516.5) | | 15.9 (13.3-18.9) |
| % of TE from animal protein 9.1 (2.0-13.1) | | 7.2 (4.5-10.7) |  | 10.9 (7.8-14.0) |
| % of TE from plant protein 4.4 (0.9-7.3) | | 4.9 (4.0-5.8) |  | 3.5 (2.7-4.4) |
| % of TE from fat | 34.0 (17.5-44.6) | 31.6 (19.0-44.9) | | 38.5 (23.9-54.6) ^a^ |
| % of TE from carbohydrates | 50.1 (33.8-67.2) | 56.7 (51.3-62.2) ^a^ | | 45.7 (39.5-47.1) |

MUFA, Monounsaturated fatty acids; PUFAs, polyunsaturated fatty acids; RE, retinol equivalent; TE, total energy. “a”, significant difference (*p* < 0.05).

**Table S2.** Extracted nutrient patterns and factor loadings identified by factor analysis in the

Black and White groups.

| **Nutrients and variance explained** | **Factors (nutrient patterns)*** | | | |
| --- | --- | --- | --- | --- |
|  | **Factor 1**  **‘Animal protein and saturated fat’** | **Factor 2**  **‘Magnesium, potassium, calcium, phosphorus and fiber’** | **Factor 3**  **‘Plant protein, B-vitamins, zinc and iron’’** | **Factor 4**  **‘Vitamin E and PUFAs’** |
| Animal protein | **0.764** |  |  | –0.314 |
| Saturated fat | **0.678** |  |  |  |
| Plant protein | –0.635 |  | **0.486** |  |
| Magnesium |  | **0.793** |  |  |
| Potassium |  | **0.769** |  |  |
| Phosphorus | 0.550 | **0.718** |  |  |
| Calcium | 0.324 | **0.610** |  |  |
| Total CHO | –0.413 |  | 0.364 |  |
| Total fibre | –0.510 | **0.520** |  |  |
| Vitamin C |  | 0.380 |  |  |
| Biotin | 0.453 |  |  |  |
| Vitamin A (RE) |  | 0.359 |  |  |
| Niacin |  |  | **0.684** |  |
| Vitamin B6 | –0.319 |  | **0.681** |  |
| Thiamine |  |  | **0.646** |  |
| Zinc |  |  | **0.597** |  |
| Total iron |  |  | **0.560** |  |
| Vitamin E |  |  |  | **0.819** |
| PUFA |  |  |  | **0.782** |
| Vitamin D |  |  |  | 0.311 |
| Percent of variances  Total 51.6% | 15.54 | 14.55 | 13.71 | 7.77 |

* Extraction Method: Principal axis factoring; Rotation method: Varimax with Kaiser normalization; Estimates from a factor analysis performed

on 20 nutrients after rotation. The loadings indicate the significance of the corresponding nutrient to the factor. The leading nutrients were

defined as loadings >0.4 for each factor, shown in bold.

CHO, carbohydrates; PUFAs, polyunsaturated fatty acids; RE, retinol equivalents.

**TABLE S3** Nutrient recommendations used for evaluating nutrient intakes of the African-PREDICT study

| **Nutrients** | **Men**  **19-30 y.o.** | **Women**  **19-30 y.o.** | **Source** |
| --- | --- | --- | --- |
|  |  |  |  |
| Total protein, g | 56 | 46 | AI |
| Calcium, mg | 800 | 800 | EAR |
| Total iron, mg | 6·0 | 8·1 | EAR |
| Magnesium, mg | 330 | 255 | EAR |
| Phosphorus, mg | 580 | 580 | EAR |
| Potassium, mg | 4700 | 4700 | AI |
| Zinc, mg | 9·4 | 6·8 | EAR |
| Copper, µg | 700 | 700 | EAR |
| Vitamin A RE, µg | 625 | 500 | EAR |
| Thiamine, mg | 1·0 | 0·9 | EAR |
| Riboflavin, mg | 1·1 | 0·9 | EAR |
| Niacin, mg | 12 | 11 | EAR |
| Vitamin B6, mg | 1·1 | 1·1 | EAR |
| Folate, µg | 320 | 320 | EAR |
| Vitamin B12, µg | 2·0 | 2·0 | EAR |
| Pantothenic acid, mg | 5·0 | 5·0 | AI |
| Biotin, µg | 30 | 30 | AI |
| Vitamin C, mg | 75 | 60 | EAR |
| Vitamin D, mg | 5·0 | 5·0 | AI |
| Vitamin E, mg | 12·0 | 12·0 | EAR |
| AI, Adequate intake ^(34, 35)^  EAR, Estimated average requirements ^(34, 35)^ | | | |
